# Supplementary material for: Short-range order in high entropy carbides
Source: Nat Commun. 2026 Feb 4;17:2362. doi: 10.1038/s41467-026-69095-8 (PMC12979665; doi:10.1038/s41467-026-69095-8)
Supplement: Supplementary file 1 — Supplementary Information [file 41467_2026_69095_MOESM1_ESM.pdf]

Supplementary Information

Short-range order in high entropy carbides

Shuguang Wei[1*], Muhammad Waqas Qureshi[1*], Jingrui Wei[1*], Longfei Liu[1*], Xuanxin Hu[1], Jianqi Xi[2], Siamak Attarian[1], Ranran Su[3], Hongliang Zhang[4], Evan Willing[5], Xudong Wang[1], Kumar Sridharan[5], Paul M. Voyles[1], John H. Perepezko[1], Izabela Szlufarska[1, #]

[1]Department of Materials Science and Engineering, University of Wisconsin–Madison, Madison, WI 53706, United States

[2]Department of Nuclear, Plasma & Radiological Engineering, University of Illinois Urbana-Champaign, Urbana, IL 61801, United States

[3]School of Nuclear Science and Engineering, Shanghai Jiao Tong University, Shanghai 200240, China

[4]Institute of Modern Physics, Fudan University, Shanghai 200433, China

[5]Department of Nuclear Engineering and Engineering Physics, University of Wisconsin–Madison, Madison, WI 53706, United States

**These authors contributed equally.*

[#]Corresponding author:

szlufarska@wisc.edu (I. Szlufarska)

I. Potential energy of simulated samples.

II. Ordering and disordering in HECs.

III. Supplementary Note 1｜Electron channeling effects.

IV. Correlation of local strain with local concentration.

V. Structure and grain size distribution of HEC-Mo.

VI. SRIM simulation of damage and ion distribution for HECs.

VII. SRIM simulation of damage and ion distribution for HECs.

VIII. Supplementary Note 2｜Cepstrum analysis

IX. XRD, EDS and XPS analysis of HECs before irradiation.

X. GIXRD profiles of HECs before and after irradiation near the (220) peak.

References

I. Potential energy of simulated samples.

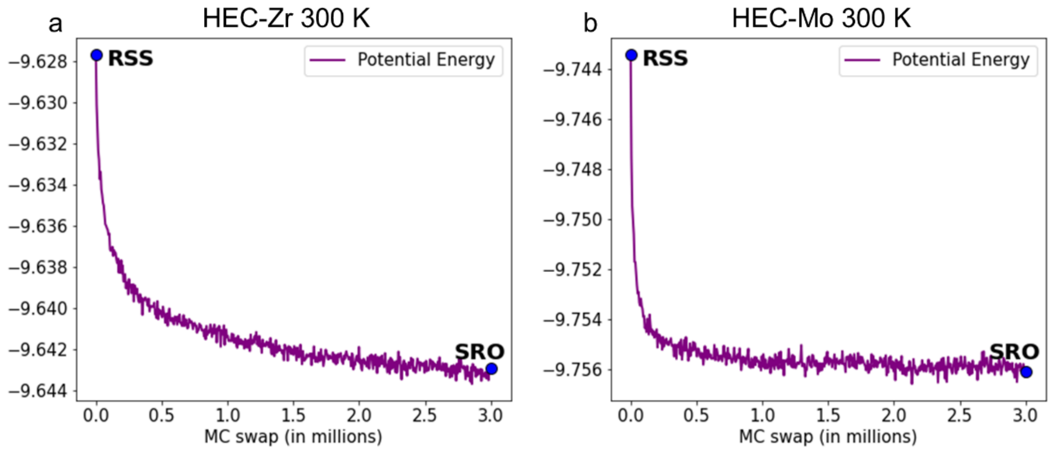

Figure S1: Potential energy of simulated samples as a function of Monte Carlo (MC) swaps. *Potential energy of HEC-Zr simulated at (a) 300 K and (b) HEC-Mo simulated at 300 K.*

II. Ordering and disordering in HECs.

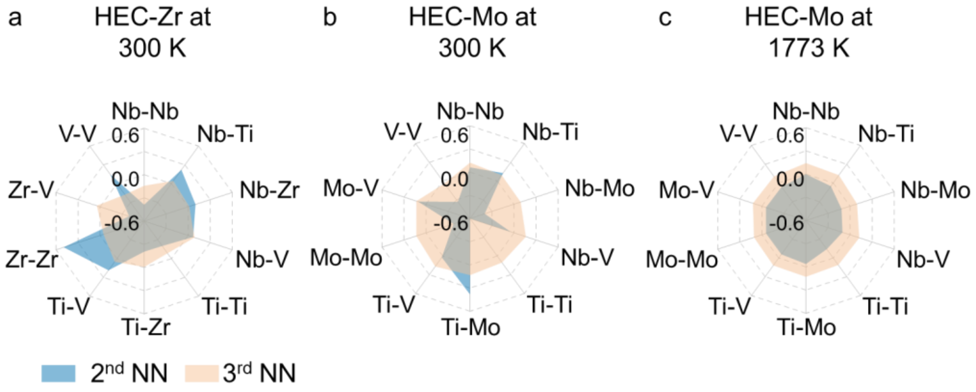

Figure S2: The Warren-Cowley parameter when considering the 2nd, and 3rd nearest neighbor (NN) metal-metal interactions within the cation sublattice. *(a) HEC-Zr at 300 K, (b) HEC-Mo at 300 K and (c) HEC-Mo at 1773 K.*

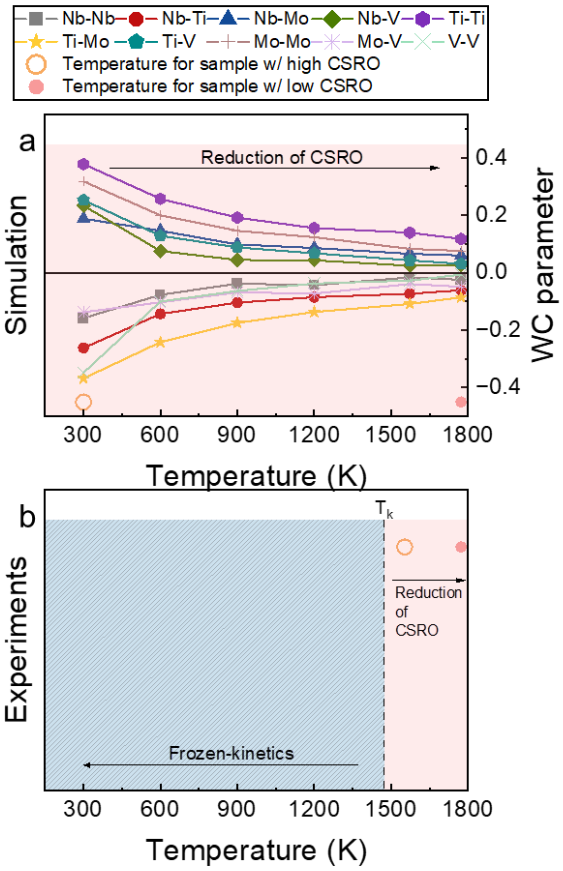

Figure S3: Degrees of CSRO in HEC-Mo identified using simulations and experiments. *(a) The 1st NN Warren-Cowley parameter in HEC-Mo as a function of temperature. CSRO is dramatically reduced after ~900 K. The annealing temperatures used in simulations to study different degrees of CSRO in HEC-Mo are labeled as open and close symbols, respectively. (b) Experimental observation of the temperature dependence of CSRO and the onset temperature of CSRO kinetics (T_k) in HEC-Mo measured using DTA. The annealing temperatures used in experiments to study different degrees of CSRO in HEC-Mo are also labeled as open and close symbols, respectively. Red shaded area represents the region where the degree of CSRO decreases with the increase of temperature. The blue shaded area represents the region where the kinetics of CSRO are frozen.*

The WC parameters of HEC-Mo as a function of temperature were calculated and plotted in Fig. S3a. The extent of CSRO decays with the increase of temperature and drops significantly after ~900 K (see Fig. S3a). DTA experiments provided information about the temperature dependence of CSRO (see Fig. 2a in the main text). The data shows that CSRO drops significantly in HEC-Mo above ~1653 K (Fig. S3b). At the same time, CSRO in experiments can form only when the temperature is higher than the kinetic temperature (T_k) at ~1473 K, since the kinetics for CSRO is frozen below 1473 K. There is a discrepancy between experimental and simulation temperatures above which CSRO is largely dissolved. It is not a concern for the analysis since the simulated systems are selected from temperatures well below and well above the temperature where CSRO becomes significantly reduced. For the system with low

CSRO, we use the temperature of 1773 K both in simulations and in experiments. However, to study samples with high CSRO, we had to choose different temperatures for simulations and experiments, and they were chosen to be 300 K and 1573 K, respectively. The reason we did not perform experiments at 300 K is because this temperature is below the experimental value of T_k.

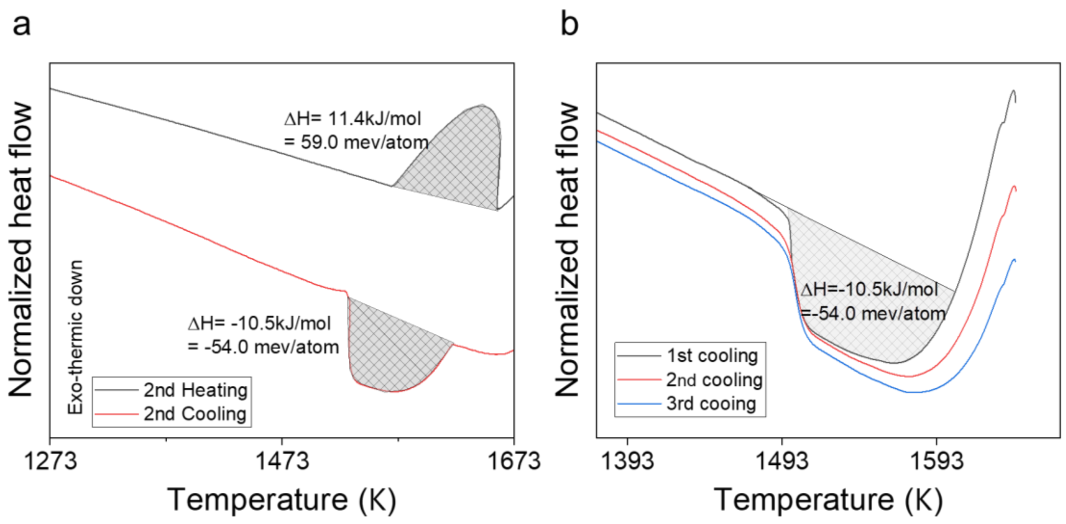

Figure S4: DTA scans of HEC-Zr heating and cooling curves. *(a)DTA heating and cooling curves of HEC-Zr during the 2nd cycle scanning. Peaks are induced by the formation and dissolution of CSRO. (b) DTA cooling curves of HEC-Zr up to 3 cycles. Nearly identical base line and comparable peak areas suggest that our DTA method bears good reproducibility. ΔH represents the enthalpy change of HEC-Zr during the formation or dissolution of CSRO. Shaded areas represent the amount of heat released and absorbed during formation and dissolution of SRO, respectively.*

III. Supplementary Note 1 | Electron channeling effects.

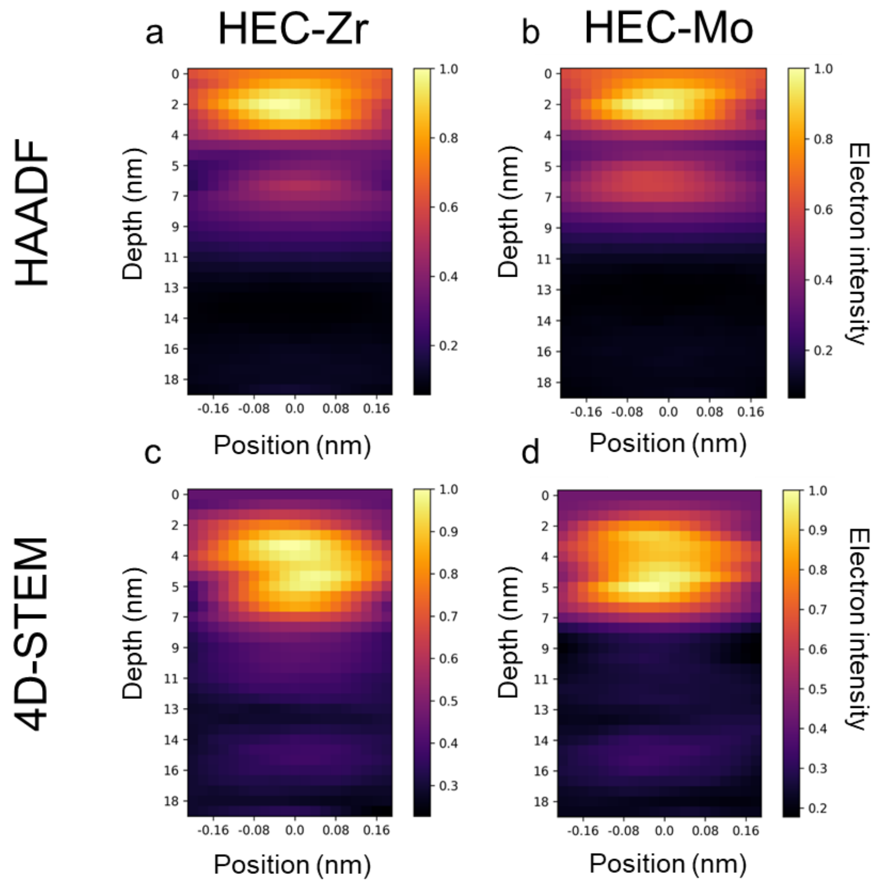

Figure S5: Electron channeling effects of HAADF and 4D-STEM. *a, b, 2D intensity profile of the STEM probe propagating through a metal column in the simulated HEC supercells when performing HAADF imaging. c, d, 2D intensity profile of the STEM probe propagating through a metal column in the simulated HEC supercells when performing 4D-STEM imaging. All intensities are normalized by the maximum value. When collecting HAADF images, atoms in the top 4 nm contribute the most to the images. However, atoms in the range of 4-10 nm contribute 50 % less than the top 4 nm and become negligible below 10 nm, which is the reason why the contrast induced by CSRO can be reflected by HAADF. When collecting 4D-STEM, atoms in the top 8 nm contribute the most. Whereas the atoms in the range of 8-18 nm contribute 60 % less than the top 8 nm and become negligible below 18 nm. Thus, strains induced by CSRO can still be interpreted by 4D-STEM.*

We performed simulations to demonstrate that the averaging effect is insignificant in our studies. More specifically, during the collection of STEM data, dynamical scattering leads to electron channeling effect. For example, when imaging zone-axis oriented crystals, the data does not reflect a simple two-dimensional projection of the structure. Instead, the on-column probe exhibits an intensity peak near the electron entrance surface (top surface), which means that atoms in a thin region near the top surface dominate the collected signals[1]. We performed simulations of electron

beam intensity along a column of atoms with a convergence semiangle of 23.4 mrad to simulate the condition of HAADF-STEM imaging (Fig. S5a, b). Intensity peaks can be observed in the region from the top surface to a depth of 4 nm for both samples (Fig. S5a, b), suggesting atoms in the top 4 nm contribute the most to our collected data. Atoms in the region extending from 4 nm to 10 nm from the surface can make contributions, but their contributions dropped by 50%. Contributions from atoms beyond 10 nm can be neglected. The depth of 4 nm where the intensity reaches the peak is comparable to the depth of field (5.9 nm) calculated by *Li L. et al.*[2], where HAADF-STEM was also employed to directly derive cation distributions in cation-disordered oxides. In summary, the atoms in the top 4 nm dominated the collected data during HAADF-STEM. And the averaging effects are most significant in the top 4 nms. Considering that the majority of CSRO domains are in the range of 1-2 nm, they can be resolved by HAADF-STEM.

Regarding the 4D-STEM, a smaller convergence angle (3 mrad) was used. The electron channeling effect under such circumstances was also explored (see Fig. S5c, d). Intensity peaks appear in the regions from the top surface to a depth of 8 nm for both samples (Fig. S5c, d), indicating atoms in the top 8 nm contribute the most to our collected data. Atoms in the region extending from 8 nm to 18 nm can make contributions, but their contributions decreased by 60 %. Contributions from atoms below 18 nm can be neglected. Overall, atoms in the top 8 nm dominated the collected data during 4D-STEM and the averaging effects are most considerable in the top 8 nm. To explore if the CSRO induced strain can still be resolved when the signals are averaged by the atoms in a relatively larger region, 4D-STEM analysis were performed on our MD/MC samples with a thickness of 7 nm, which is close to the dominant region of 8 nm from top surface. According to our analysis, the highest strains measured are 6.1%, 4.6% and 4.4% for HEC-Zr, HEC-Mo and HEC-Mo annealed at 1773 K, respectively. The trend of the highest strains among different samples agrees well with our experimentally measured strain trend calculated directly from 4D-STEM, illustrating that the strain induced by CSRO can be resolved by 4D-STEM and the averaging effects in the top 8 nm can be neglected.

IV. Correlation of local strain with local concentration.

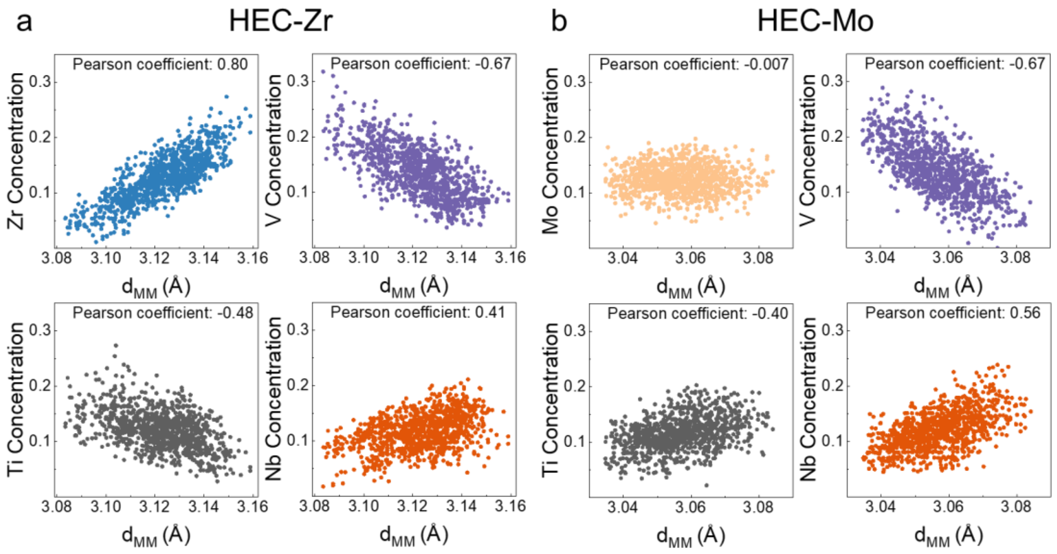

Figure S6: Correlation of local strain with local concentration. *scatter plot of local concentration of the 4 cations with respect to local mean cation bonding length (d_{MM}) for HEC-Zr (a) and HEC-Mo (b). The sub-volume is approximately the illuminated volume of the experiment STEM probe. The data is from 30 × 30 grid sampling points over a 3×3 nm area of MD models. Pearson correlation function was calculated for each pair of variables, a value close to 1 indicates strong correlation, a value closer to -1 indicates strong anti-correlation, and 0 means no correlation.*

V. Structure and grain size distribution of HEC-Mo.

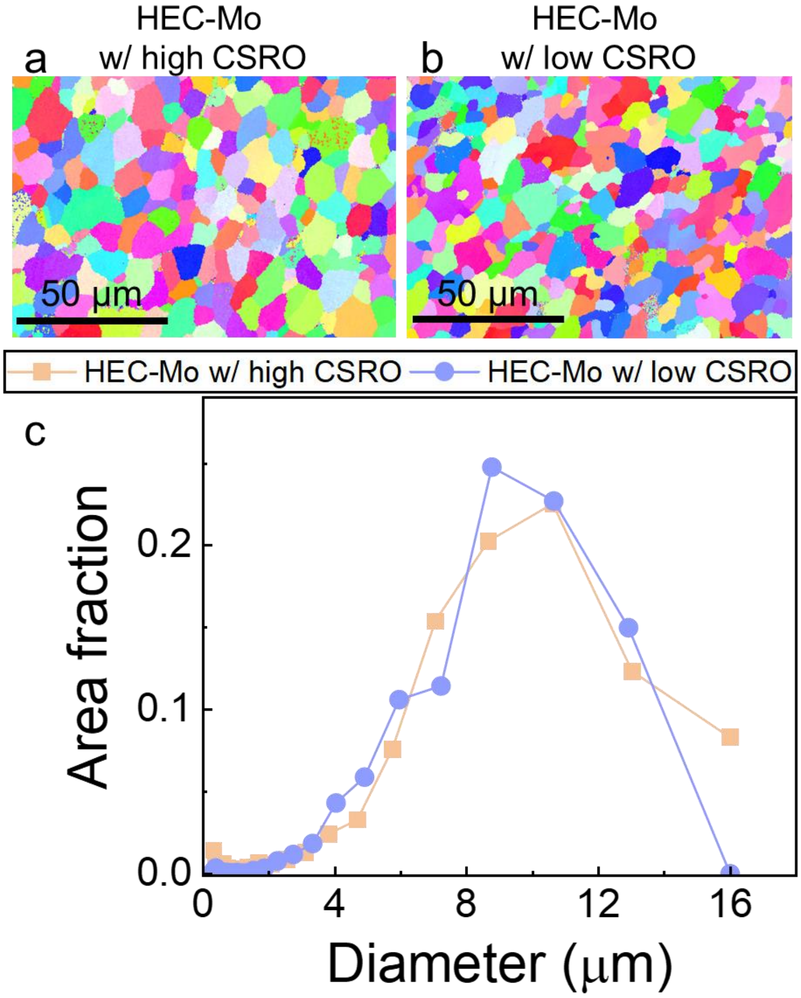

Figure S7: Structure and grain size distribution of HEC-Mo with high CSRO (as-synthesized) and HEC-Mo with low CSRO (annealed at 1773 K). *Electron backscatter diffraction (EBSD) map of HEC-Mo with high CSRO (a) and HEC-Mo with low CSRO (b) before irradiation. (c) Histogram of grain size in HEC-Mo with high CSRO and HEC-Mo with low CSRO measured using EBSD.*

VI. SRIM simulation of damage and ion distribution for HECs.

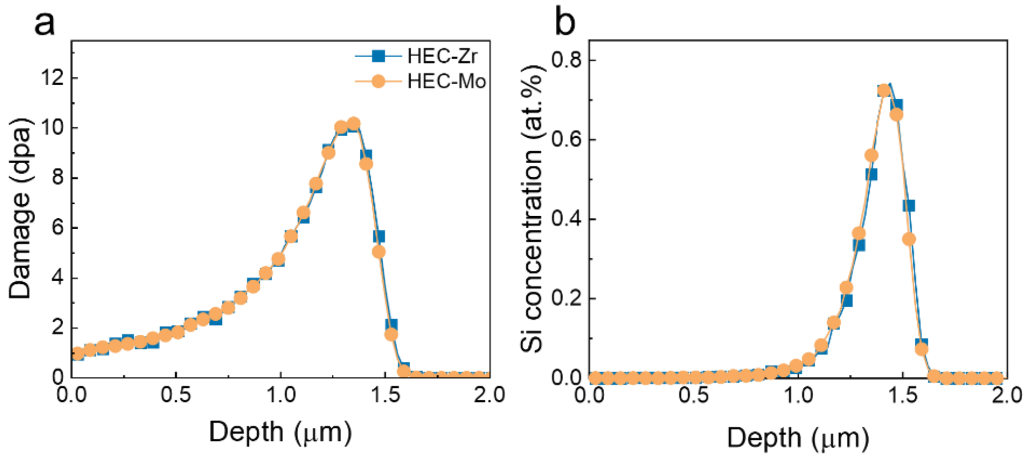

Figure S8: SRIM calculation of radiation induced damage and Si ion concentration in HECs. *a. Irradiation damage in dpa versus depth profile for HEC-Zr and HEC-Mo at the fluence of 1.96×10^{16} ions·cm^{-2}. b. Si concentration as a function of depth profile for HEC-Zr and HEC-Mo.*

VII. SRIM simulation of damage and ion distribution for HECs.

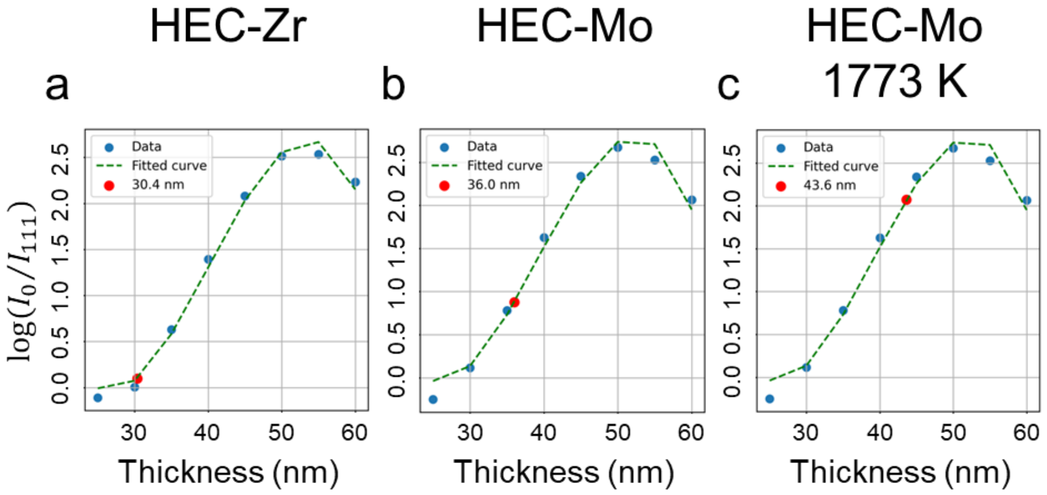

Figure S9: Thickness of regions for 4D-STEM analysis. *Thickness was measured using the ratio of intensities of the transmitted beam (**I_0**) and a {111} diffracted beam (**I_{111}**), which depends on thickness due to dynamical scattering[3]. The ratio was compared to simulations using multislice methods with different thickness. Thickness of HEC-Zr (a) was measured to be 30.4 nm, HEC-Mo (b) was measured to be 36.0 nm and HEC-Mo annealed at 1773 K (c) was measured to be 43.6 nm.*

VIII. Supplementary Note 2 | Cepstrum analysis

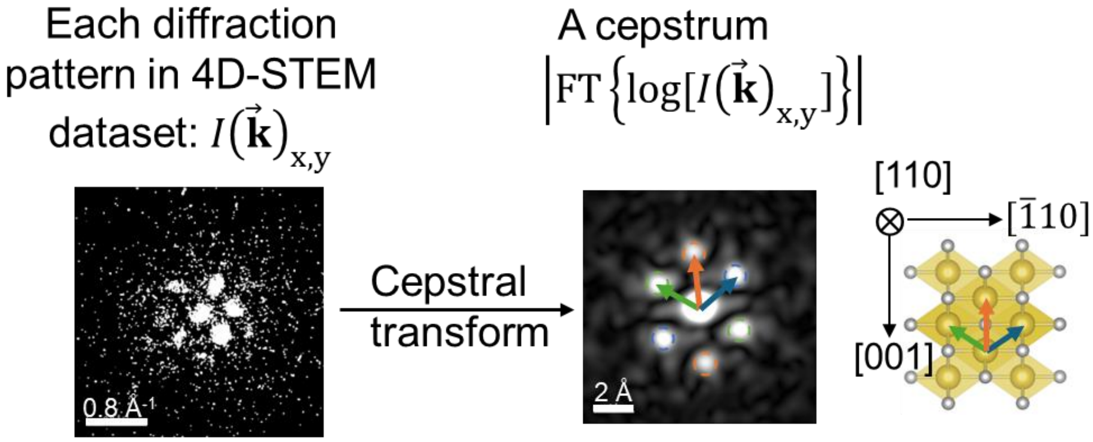

Figure S10: Illustration of cepstrum analysis method. The graphical representation of atomic structure is generated using VESTA[4]. *The electron diffraction pattern collected during the 4D-STEM (left) was transformed to a cepstrum (middle) and the direction of each peak was illustrated in the atomic structure (right).*

We applied cepstral transform to 4D-STEM dataset by taking the Fourier spectrum of the natural log of the diffraction patterns. Hanning window and zero padding was used to improve the cepstrum quality and sampling. Strain measurement was accomplished by locating the $\{\bar{1}10\}$ peaks (labeled as green and blue) to their maxima intensity pixel.

For local distortion measurements in cepstrum, the equation of cepstrum by Saran Pidaparthy *et al*[5], where the diffraction intensity is expressed as follows:

$$\langle I(\mathbf{k})\rangle_r = |f(\mathbf{k})|^2 \cdot (N \cdot S(\mathbf{k}))$$

Here, N is the total number of probed atoms and $S(\mathbf{k})$ is the static structure factor, which for a uniform and isotropic system, is related to the pair distribution function $g(\mathbf{r})$ directly by the following expression:

$$S(\mathbf{k}) = 1 + \rho(\int \exp(-i\mathbf{k}\cdot\mathbf{r})g(\mathbf{r})d\mathbf{r}),$$

where ρ is the atomic density. Then, the Fourier spectrum of $\langle I(\mathbf{k})\rangle_r$ can be simplified as follows:

$$|\mathcal{F}\{\langle I(\mathbf{k})\rangle_r\}| = |\mathcal{F}\{|f(\mathbf{k})|^2\} \otimes [N\delta(\mathbf{r}) + N\rho(g(\mathbf{r})-1)]|$$

Here, we assume that the difference in the first term of the convolution is negligible between different HEC specimens, as the atomic scattering power of the elements has only very small differences. The cepstrum peaks thus inherit the characteristics of pair distribution function peaks. We measured the cepstrum peak width using a 2D Gaussian fit and used the sigma value.

IX. XRD, EDS and XPS analysis of HECs before irradiation.

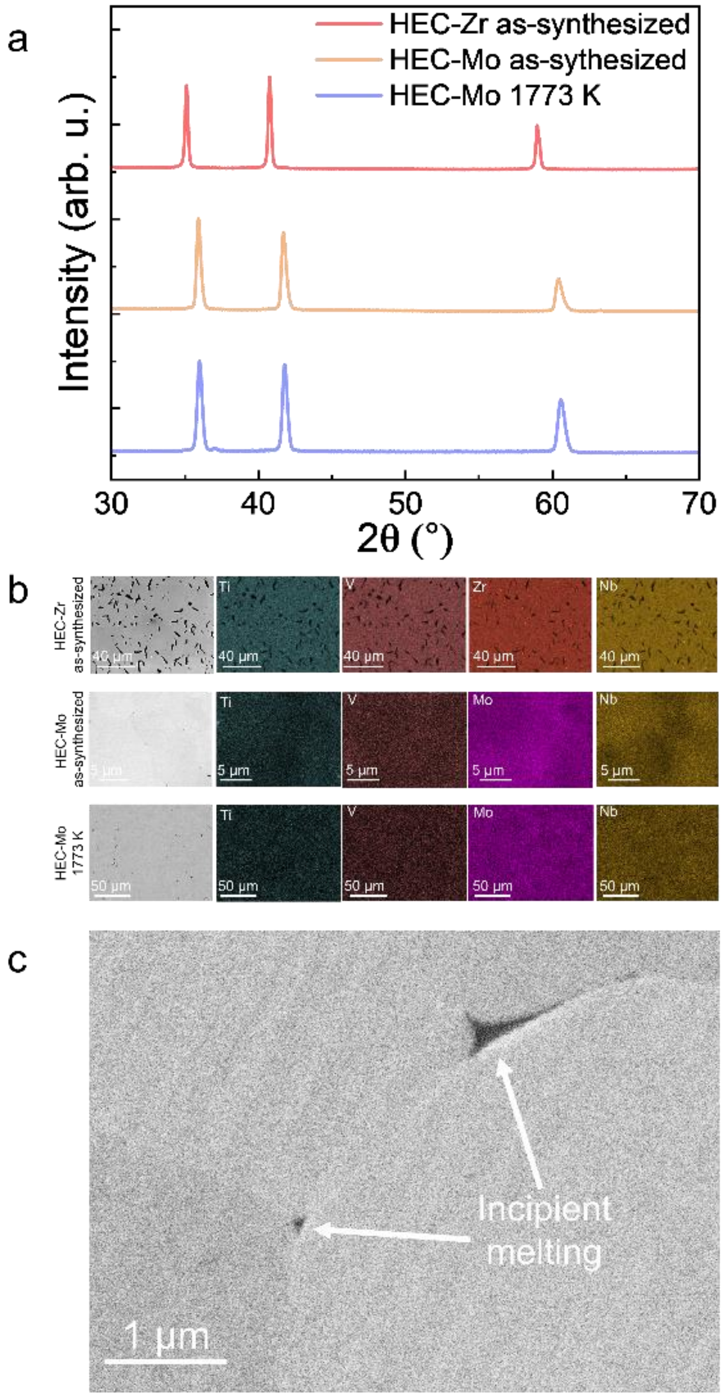

Figure S11: Characterization of HECs before irradiation. *(a), XRD of HEC-Zr as-synthesized, HEC-Mo as-synthesized and HEC-Mo heat-treated. (b), EDS area scan on as-synthesized HEC-Zr, as-synthesized HEC-Mo and HEC-Mo annealed at 1773 K. XRD profiles indicating that all samples exhibited FCC single phase and EDS area scans suggest uniform distribution of cations. (c) SEM image of examples for the formation of pockets at grain boundary junctions in HEC-Mo that are characteristics of the presence of molten zones as indicated by arrows.*

Table S1. XPS measured composition for HECs

**Sample**	**HEC-Zr**					**HEC-Mo**				
**Element**	Ti	V	Zr	Nb	C	Ti	V	Mo	Nb	C
**Orbital**	2*p*	2*p*	3*d*	3*d*	1*s*	2*p*	2*p*	3*d*	3*d*	1*s*
**Area**	2.26	3.54	2.69	2.38	1.74	2.04	3.97	2.73	2.36	1.27
**at.%**	9.33	11.97	12.49	9.48	56.72	10.65	12.98	11.91	11.87	52.60

X. GIXRD profiles of HECs before and after irradiation near the (220) peak.

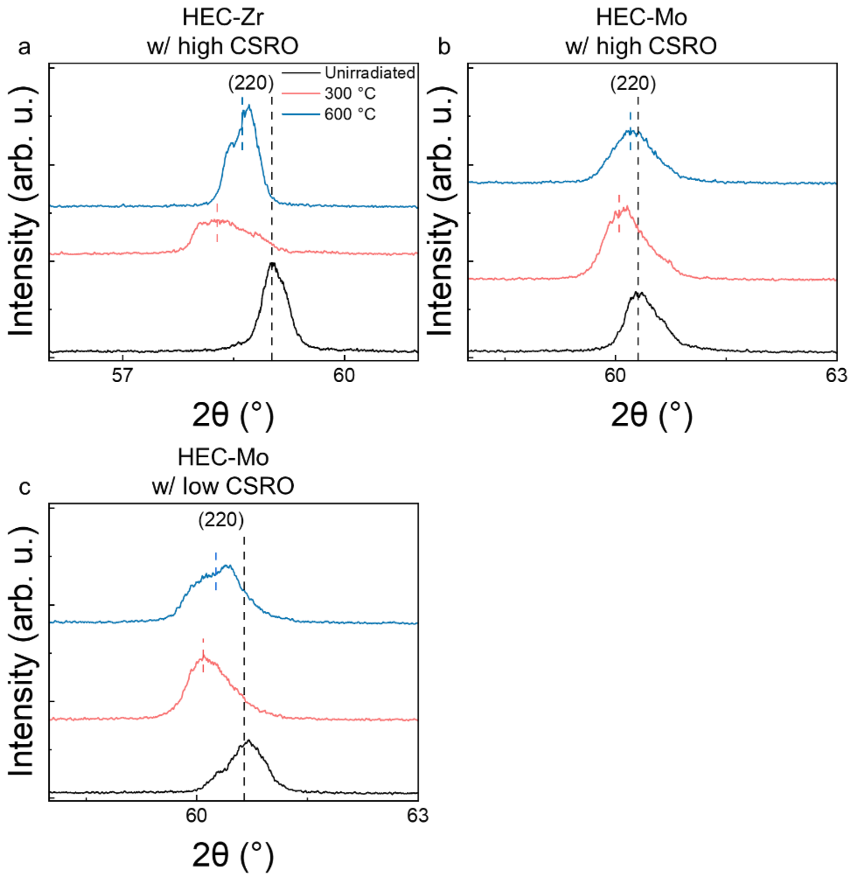

Figure S12: GIXRD profiles of HECs before and after irradiation near the (220) peak. *XRD profiles of HEC-Zr with high CSRO (as-synthesized) (a), HEC-Mo with high CSRO (as-synthesized) (b) and HEC-Mo with low CSRO (annealed at 1773 K) (c).*

References

1. Voyles, P. M., Muller, D. A. & Kirkland, E. J. Depth-Dependent Imaging of Individual Dopant Atoms in Silicon. *Microscopy and Microanalysis* 10, 291–300 (2004).
2. Li, L. *et al.* Atomic-scale probing of short-range order and its impact on electrochemical properties in cation-disordered oxide cathodes. *Nat Commun* 14, 7448 (2023).
3. Voyles, P. M., Muller, D. A., Grazul, J. L., Citrin, P. H. & Gossmann, H.-J. L. Atomic-scale imaging of individual dopant atoms and clusters in highly n-type bulk Si. *Nature* 416, 826–829 (2002).
4. Momma, K. & Izumi, F. *VESTA 3* for three-dimensional visualization of crystal, volumetric and morphology data. *J Appl Crystallogr* 44, 1272–1276 (2011).
5. Pidaparthy, S., Ni, H., Hou, H., Abraham, D. P. & Zuo, J. M. Fluctuation cepstral scanning transmission electron microscopy of mixed-phase amorphous materials. *Ultramicroscopy* 248, 113718 (2023).
